# Progress in Surgery

**Published:** 1900-03-10

**Authors:** 


### Progress in Surgery.

### SURGERY OF THE STOMACH.

**Perforated Gastric Ulcers.**—Barker[1] reports 12 consecutive cases of this affection, all of which were as nearly as possible treated under the same conditions. The immediate results were that five out of 12 were saved. Two of the fatal cases lived 15 and 14 days respectively, the first dying from sub-phrenic abscess and consequent pleuro-pneumonia, the second from bleeding into the stomach from the ulcer, the conditions following the operation being otherwise favourable. In all the 12 cases mopping out of the abdominal cavity was preferred to flushing. Thorough sponging beneath the diaphragm was important. Drainage was only employed in one case, and was then shown to have been unnecessary. In none of the other cases would it have improved the patient's chances. A vertical incision was made in all of the cases, which was enlarged laterally in one. Sutures of hard silk were used. Free gas was almost always present in the abdominal cavity. Ten of the cases were females and two were males. Both the male cases died. In only three of the series was there a previous history of vomiting, and in only two was there definite hematemesis. Nothing seemed to affect the issue of the case more than the time which had elapsed between the perforation and the operation. The average length of time in the five successful cases was $11\frac{3}{4}$ hours, that in the seven fatal cases $30\frac{1}{2}$ hours. Collapse was absent in six out of the 12 cases. Haward[2] agrees that flushing the abdomen is generally unnecessary in these cases, for flushing carries septic matter to remote parts. Bidwell[3] summarises the various complications of gastric ulcer requiring surgical interference. These complications are—(1) Perforation into the general peritoneal cavity; (2) perforation of the posterior wall of the stomach and sub-phrenic abscess; (3) hæmorrhage; (4) dilatation from pyloric stenosis, the result either of cicatricial contraction within, or of adhesions without, the stomach. Perforation into the general peritoneal cavity is variously estimated by statistics as taking place in from 1 to 12 per cent. of all cases of ulcer. The most important factor in success is early operation. In the author's cases 33 out of 55 recovered after operation. All cases operated on within 12 hours (13 in number) were successful; of the 14 operated upon between 12 and 24 hours one-half were cured; and of the 15 operated upon after a longer interval than 24 hours only 5, or one-third, were cured. In cases of ulcer associated with very severe or repeated hæmorrhage, the author recommends that the stomach should be opened and emptied of its contents. If an ulcer can be seen, and its base be not contracted by adhesions outside, it should be surrounded by a purse-string suture so as to constrict it. If the ulcer be adherent gastro-enterostomy should be performed. The general results of operation for hæmorrhage are not satisfactory; in 21 cases there was a mortality of 61·9 per cent. For old-standing cases with dilatation from pyloric spasm, or actual pyloric ulceration and obstruction, either a gastro-enterostomy or a pyloroplasty should be performed. Petersen[4] publishes the results of 50 operations performed for simple ulcers of the stomach and their sequelæ. Pyloric stenosis with great motor deficiency, accompanied by emaciation and a permanent diminution of the quantity of urine, is an absolute indication for operative interference. When internal treatment fails, severe atonic motor deficiency, profuse hæmorrhages, and severe gastralgia with uncontrollable vomiting, whether dependent on recent ulceration, cicatrices of ulcers, perigastritis, or adhesions, also demand surgical treatment. Since 1895 the mortality of these operations has declined from 22 per cent. to 2·3 per cent. This great improvement is to be explained by (1) the operation having been performed earlier, when the patients were in better condition; (2) greater experience in the choice of cases, and especially the substitution whenever possible of gastro-enterostomy or pyloroplasty for excision and resection; and (3) improved technique, especially the use of Murphy's button, which has reduced the time required for the operation from three-quarters to a quarter of an hour, allowed the patients to be fed by the mouth directly after the operation, lessened the chance of subsequent pneumonia, and abolished the danger of the formation of a "spur." The effect of gastro-enterostomy on the gastric function is of interest: (1) Dilatation diminishes, though the stomach is seldom reduced to its original size; (2) the motor function is, as a rule, completely restored; (3) the percentage of hydrochloric acid sinks; and (4) lactic acid disappears. Cases of successful operation for perforating gastric ulcer are reported by Adamson,[5] and Davey and Eve.[6]

**Pyloroplasty.**—Koeppelin[7] reports a third case of the modification of Heineke and Mikulicz's operation, known as sub-mucous pyloroplasty. The horizontal wound along the pylorus is made through the two outer coats, serous and muscular, of the bowel, and through the cicatrix if there be one. The exposed and unopened mucosa at once bulges like a hernia. The divided coats are then united vertically as in ordinary pyloroplasty. All three cases of the sub mucous operation have proved successful. The author maintains that this modification is safer than the older pyloroplasty. Monson[8] has performed the ordinary operation 21 times in cases of pyloric stricture without a death. The indications for operation are: (1) Prolonged dyspeptic symptoms, and especially frequent vomiting. (2) Dilatation of the stomach (always present) and the presence of a movable and painful tumour, a little to the right of, and above, the umbilicus. The operation is simple, and is not followed by recurrence of the stricture. Richardson[9] also reports three successful cases of pyloroplasty for stricture of the pylorus. In some cases of chronic cholecystitis, and other inflammatory diseases near the pylorus, peritoneal adhesions may produce great disturbance of nutrition, even though there be no organic disease of either pylorus or duodenum. In some cases a partial excision must also be performed, in order that the longitudinal incision may easily and safely be sutured in a transverse direction. He condemns digital dilatation of benign stricture of the pylorus as unsatisfactory and useless.

**Gastro-enterostomy.**—Perman[10] gives a record of 42 cases of this operation; 27 of these were for carcinoma of the pylorus, and of these 16 recovered and 11 died. Wölfler's method (anterior wall) was adopted at first, but the occurrence of severe bile vomiting in two cases led to the employment of Von Hacker's method (posterior wall), which, with continued sutures, the author strenuously advises as the best operation. Pylorectomy, according to the statistics of Haberkants, Carles, and Fantinos (1898), only yields about 30 per cent. of recoveries, and should, Perman thinks, only be used for very early cases of carcinoma.

**Injection of Fluids and Air into the Stomach from the upper part of the Œsophagus.**—Krönig[11] recommended that in carcinomatous and cicatricial changes, and still more in recent corrosion of the œsophagus, the use of the sound should be preceded by an injection of 20—30 c.cm. of warm olive oil by means of a soft Nelaton's catheter, about 15 cm. long, the eye of which reached to about the limit between the first and second upper fourths of the œsophagus, after which the tube can be passed into the stomach without danger or pain. Finding that the oil so injected passed contracted points with a certain ease, and without any act of swallowing on the part of the patient, and finding that watery fluids also passed into the stomach, he proceeded to inject air for diagnostic inflation of the stomach from the same height. It is an undoubted advantage both to the patient and the physician to avoid passing the sound into the stomach. Krönig now employs this method for introducing food, especially in strictures that the sound cannot pass without difficulty and danger, and attaches much importance to its mechanical effect in keeping open or even dilating the passage. The proceeding is so simple, and causes so little distress, that the patient after a few days can introduce the catheter himself, and, with a view to its gentle mechanical effect, it may be commenced as soon as he perceives any difficulty in swallowing.

[1] Lancet, Dec. 16, 1899, p. 1668. [2] Ibidem, p. 1669. [3] Med. Chron., Oct., 1899, p. 37; and Amer. Jour. Med. Sci., Sept., 1899. [4] Brit. Med. Jour., Sept. 30, 1899, Epitome No. 257; and Deut. Med. Woch., June 15 and 22, 1899. [5] Scottish Med. and Surg. Jour., Dec., 1899, p. 513. [6] Lancet, Jan. 20, 1900, p. 155. [7] Brit. Med. Jour., Jan. 6, 1900, Epitome No. 7; and Lyons Méd., Sept. 24, 1899. [8] Glasgow Med. Jour., Jan., 1900, p. 72; and Edin. Med. Jour., Aug., 1899. [9] Brit. Med. Jour., Jan. 13, 1900, Epitome No. 22; and Boston Med. and Surg. Jour., Nov. 30, 1899. [10] Med. Chron., Dec., 1899, p. 185; and Hygiea (Stockholm), Oct., 1899. [11] Brit. Med. Jour., Nov. 25, 1899, Epitome No. 405; and Deut. Med. Woch., 1899, No. 44.

